# The 70 kDa Heat Shock Protein Assists during the Repair of Chilling Injury in the Insect, *Pyrrhocoris apterus*

**DOI:** 10.1371/journal.pone.0004546

**Published:** 2009-02-20

**Authors:** Vladimír Koštál, Michaela Tollarová-Borovanská

**Affiliations:** 1 Biology Centre ASCR, Institute of Entomology, České Budějovice, Czech Republic; 2 University of South Bohemia, Faculty of Science, České Budějovice, Czech Republic; Universidade de Brasília, Brazil

## Abstract

**Background:**

The *Pyrrhocoris apterus* (Insecta: Heteroptera) adults attain high levels of cold tolerance during their overwintering diapause. Non-diapause reproducing adults, however, lack the capacity to express a whole array of cold-tolerance adaptations and show relatively low survival when exposed to sub-zero temperatures. We assessed the competence of non-diapause males of *P. apterus* for responding to heat- and cold-stresses by up-regulation of 70 kDa heat shock proteins (Hsps) and the role of Hsps during repair of heat- and cold-induced injury.

**Principal Findings:**

The fragments of *P. apterus* homologues of Hsp70 inducible (PaHsp70) and cognate forms (PaHsc70) were cloned and sequenced. The abundance of mRNA transcripts for the inducible form (qPCR) and corresponding protein (Western blotting) were significantly up-regulated in response to high and low temperature stimuli. In the cognate form, mRNA was slightly up-regulated in response to both stressors but very low or no up-regulation of protein was apparent after heat- or cold-stress, respectively. Injection of 695 bp-long *Pahsp70* dsRNA (RNAi) caused drastic suppression of the heat- and cold-stress-induced *Pahsp70* mRNA response and the up-regulation of corresponding protein was practically eliminated. Our RNAi predictably prevented recovery from heat shock and, in addition, negatively influenced repair of chilling injuries caused by cold stress. Cold tolerance increased when the insects were first exposed to a mild heat shock, in order to trigger the up-regulation of PaHsp70, and subsequently exposed to cold stress.

**Conclusion:**

Our results suggest that accumulation of PaHsp70 belongs to a complex cold tolerance adaptation in the insect *Pyrrhocoris apterus*.

## Introduction

Insects evolved impressive strategies for survival at sub-zero body temperatures [1]. Many species extensively supercool their body fluids and thus avoid freezing [2], others concentrate solutes in body fluids by desiccation and, consequently, also avoid freezing [3], and still others tolerate ice formation in extra-cellular spaces [4]. A whole array of physiological adjustments counteract the damaging effects of low temperatures, *e.g.* accumulation of low molecular weight cryoprotectants [5], synthesis of antifreeze proteins [6], and remodeling of cellular membranes [7]. It has been proposed recently that heat shock proteins (Hsps) also contribute to the cold tolerance of insects [8].

The Hsps approximately 70 kDa in molecular mass represent one of five major Hsp families (small Hsps, 60, 70, 90 and 100 kDa Hsps) [9], [10]. Hsp70 proteins are found in all organisms and their structure and function are highly conserved [11]. Originally, they were discovered as being induced by heat shock, *i.e.* a brief exposure to non-lethal temperatures above the optimum for growth and development [12]. Later, it was found that a wide array of environmental stresses elicit a similar up-regulation response [13]. In addition to inducible forms, the Hsp70 family also includes constitutive forms – heat shock cognates (Hsc70). While the inducible forms assist the proteins in attaining their native conformation after partial denaturation during environmental stress, the cognates participate in various processes in an unstressed cell, such as folding of proteins after translation or membrane translocation [10], [14], [15].

Up-regulation of *hsp70* mRNA levels in response to low temperature was reported in several insect species: various fruit flies, *Drosophila sp.* [16], [17] including *D. melanogaster* [18]; adult potato beetles, *Leptinotarsa decemlineata* [19]; pupae of the onion fly, *Delia antiqua* [20]; and adults of the fly *Liriomyza huidobrensis* [21]. Relatively high levels of mRNA transcripts for heat shock proteins were also detected in diapause insects during overwintering [22], [23], [24] and in the larva of an Antarctic midge, *Belgica antarctica* [25]. Such observations led the authors to speculate on the role of Hsps in insect cold tolerance. Current evidence suggests, however, that mRNA levels provide little information on protein abundance and activity [26] and that detailed functional studies are needed to elucidate the influence of candidate genes on phenotype. Up-regulation of Hsps at the protein level was verified in the fruit flies exposed to cold [27], [28], and in the pupae of flesh fly, *Sarcophaga crassipalpiss* during diapause [29]. To our knowledge, the only direct evidence obtained so far for positive role of heat shock proteins in insect cold tolerance is that by Rinehart et al. [8] who injected the *hsp23* and *hsp70* dsRNAs (double-strand RNAs) into the pre-diapause larvae of *S. crassipalpis*, and observed a dramatic decrease of mRNA levels in pupae and a significant loss of their cold tolerance.

The main objective of this study was to assess the role of Hsp70 in cold tolerance of the adult linden bug, *Pyrrhocoris apterus* (Insecta, Heteroptera). Linden bug is one of the model insects used for the investigation into mechanisms of supercooling, cold tolerance and repair of chilling injury [7], [30]–[36]. Here we report on cloning the fragments of inducible and cognate forms of 70 kDa heat shock proteins in *P. apterus*. Further, we show that the mRNA and protein levels of the inducible form are significantly up-regulated in response to high and low temperature stimuli, and use RNAi to suppress the up-regulation responses. We demonstrate that RNAi suppression of Hsp70 levels completely prevents recovery from heat shock and negatively affects repair of chilling injury. Cold-induced expression of Hsp70 thus appears as an important component of a complex adaptive syndrome of cold tolerance in *P. apterus*.

## Methods

### Insects

The adults of the brachypterous wing-form of *P. apterus* were collected in the field during spring of 2007 near České Budějovice, Czech Republic. Laboratory colony was maintained [37] at a constant temperature of 25°C and a long day photoperiod of 18-h light : 6-h dark, which promotes direct development without reproductive diapause. F_2_ and F_3_ males, 1–2 weeks after the adult moult, were used for all experiments. Males do not significantly differ from females in their cold tolerance [33], [36], and we verified in our preliminary experiments that the sexes also do not differ in heat shock tolerance, and Hsp expression. Avoiding females allowed us to eliminate potential variation caused by dramatic cyclical changes in female physiology in connection with the cycles of egg production, mating and oviposition [38].

### Cloning and sequencing

Structural homologues of the genes encoding for 70 kDa heat shock proteins were targeted using the standard method of reverse transcription from the total RNA extracted from heat shocked *P. apterus* adults (1 h after the exposure to +41°C for 1 h). The resulting cDNA was used as a template for PCR with degenerate oligonucleotide primers designed on the basis of known sequences from other insects (upper primer, 5′-TGGGCGGCGAGGAYTTYGAYAA-3′; lower primer, 5′-AACGTGCCCAGCAGGTTRTTRTCYTT-3′). Purified PCR products were inserted into the pGEM-T Easy plasmids (Promega) and amplified by culturing in JM 109 Competent Cells (Promega). Plasmid DNA was then isolated and sequenced using BigDye Terminator v1.1 Cycle Sequencing Kit and ABI Prism 377 DNA Sequencer (Applied Biosystems). 3′-RACE technique was used to characterize the 3′-ends of cDNAs. Two sequences were obtained, named *Pahsp70* (inducible form) and *Pahsc70* (cognate form), and deposited in NCBI GenBank under accession numbers FJ386397 and FJ386398, respectively.

### RNA extraction and qRT-PCR

Abdominal fat bodies were dissected from five males and pooled to obtain one sample. Samples were stored in the RNAlater (Qiagen) at −20°C until analysis. Total RNA was extracted using RNA Blue kit (Top-Bio, Czech Republic) and the concentrations were leveled exactly to 1 µg µL^−1^ by DEPC-treated water in all samples. Five µg of total RNA was then used for a first strand cDNA synthesis using Reverse Transcription System (Promega). Relative level of mRNA transcripts for target genes was measured by quantitative real-time PCR (qPCR) using the Rotor Gene RG 3000 PCR Cycler (Corbett Research, Australia) and Hot Start version of TaKARa ExTaq DNA polymerase. PCR reactions were primed with pairs of gene-specific oligonucleotides: *Pahsp70* upper primer, 5′-CTGTGCCGATCTCTTCAGGTCAACT-3′; *Pahsp70* lower primer, 5′-GGGAGCTTTGGTCACCGCTGAGTATG-3′; *Pahsc70* upper primer, 5′-TGGAAAAGTGCAACGAAGTCATC-3′; *Pahsc70* lower primer, GGCATACCTCCAGGCATACCA-3′. Each sample was run as a technical doublet of PCR reactions for target gene and a doublet for reference gene, *Rp49* (primed with upper primer, 5′-CCGATATGTAAAACTGAGG*AGAAAC-3′; lower primer 5′-GGAGCATGTGCCTGGTCTTTT-3′). The asterisk in the *Rp49* upper primer sequence shows known position of intron which prevents annealing of this primer to genomic DNA. We verified in a preliminary test that our total RNA samples were not contaminated with genomic DNA by runing the cDNA synthesis reactions with and without AMV reverse transcriptase. In the later case, there was no product of subsequent PCR reactions with the *Pahsp70* and *Pahsc70* gene specific primers. Relative quantification of a target gene to a reference gene was done according to Pfaffl [39].

### RNAi

The cDNA prepared from heat shocked insects (+41°C/1 h) was used to amplify two complementary DNA templates for dsRNA synthesis (using TaKaRa ExTaq polymerase, annealing at 55°C, 35 cycles, Biometra T300 Thermocycler). Primers specific to *Pahsp70* were constructed with the addition of a 23 nucleotide T7 sequence to the 5′ end of each primer. The primers amplified bases 505 through 1154 of the *Pahsp70* sequence, resulting in a 695 bp fragment. DNA templates (1 µg of DNA per reaction) were used for synthesizing two complementary single-strand ssRNAs using T7 Megascript kit (Ambion) (incubation at 37°C for 4 h). After DNase treatment to remove the template, the reaction was precipitated by LiCl, washed with 70% ethanol, dried and dissolved in sterile injection buffer (1.4 mM NaCl, 0.07 mM Na_2_HPO_4_, 0.03 mM KH_2_PO_4_, 4 mM KCl, pH adjusted to 7.4). After measuring the concentration, equal amounts of two complementary ssRNAs were mixed and incubated at 95°C for 2 min followed by 70°C for 15 min and slow cooling to room temperature. Final concentration of dsRNA was adjusted to 5 µg µL^−1^.

The dsRNA for *ß*-galactosidase [40] or injection buffer alone were applied as controls. A Hamilton syringe (10 µL) was used to inject 2 µL of dsRNA or injection buffer (blank treatment) into the adult males. The needle was inserted between two abdominal tergites, close to the pleura, in parallel to the longitudinal body axis. The injected individuals showed no signs of injury and there was 100% survival of the treatment. They were allowed to recover for 2 days after the injection (our preliminary testing showed that the maximum RNAi effect develops within 2–4 days after the dsRNA injection).

### Western blotting

Proteins were extracted from the abdominal male fat bodies (3 fat bodies were pooled to obtain one sample) after homogenization in 500 µL buffer consisting of 100 mM Tris-HCl, pH 8.0; 15 mM mercaptoethanol and 1 mM EDTA. After centrifugation at 22,000 g/20 min/ 4°C, sucrose was added (0.3 M) to stabilize the proteins during storage at −80°C. Total protein concentrations were measured by the bicinchoninic acid protein assay. Proteins were separated on a 7.5% PAGE-SDS gel (Owl vertical apparatus; 4% stacking gel; running buffer: 0.025 M Tris, 0.192 M glycine, 1% SDS; constant 50 V for 30 min followed by 150 V for 1 h). Equal amounts (20 µg) of heat denatured proteins (60°C for 5 min) were loaded into each well. The proteins were transferred to the nitrocellulose (0.4 µm) membrane using the Owl semidry blotter (transfer buffer: 0.025 M Tris; 0.192 M glycine, 20% methanol, pH 8.3; constant current 1.2 mA/cm^2^ for 90 min). After briefly washing the membrane in TBST buffer, it was incubated in blocking solution for 1 h [0.5% skimmed milk (Difco) in TBST]. The membrane was hybridized overnight at 4°C with mouse monoclonal anti-heat shock protein 70 primary antibody (clone BRM-22, Sigma) diluted to 0.4 µg mL^−1^ in TBST. According to the manufacturer, this antibody recognizes both inducible and cognate forms of Hsp70 protein in various vertebrate species and also in *D. melanogaster*. In *P. apterus*, this antibody hybridized with two protein bands of approximately 70 kDa molecular mass. We have verified in preliminary tests that these bands correspond to PaHsp70 and PaHsc70 proteins by specifically quenching them with RNAi directed against *Pahsp70* mRNA (described above) and *Pahsc70* mRNA, respectively (data not shown). After thorough washings in TBST, the membrane was hybridized for 1 h with ImmunoPure Peroxidase conjugated goat anti-mouse IgG (H+L) secondary antibody (Pierce) diluted to 1 ng mL^−1^ in TBST. Washed membrane was finally incubated for 5 min in 5 mL of Supersignal West Dura solution (Pierce) to visualize the hybridization signal. After photographing the membrane using the Luminiscent Image Analyzer LAS 3000 (Fujifilm), the hybridization signal was quantified by densitometry software QuantiScan (Biosoft).

### High- and low-temperature survival and cross-tolerance

Groups of 10 insects were exposed to high or low temperatures in plastic tubes (10×60 mm) that were either placed in a Bioblock Polystat, Huber water bath (high temperature, +45°C for 3 h) or inserted into holes drilled in an aluminium block, which was situated inside the low temperature incubator F34-ME, Julabo (low temperature, −5°C for 5 days). This particular dose of cold stress (combination of temperature and duration) was selected based on our earlier survival experiments [32], [36]. After this dose of cold stress, direct mortality remained relatively low but a high proportion of insects showed signs of chilling injury. A temperature of −5°C is safely above the temperature of crystallization of body fluids, which is −9.4±0.4°C [32]. The injury which develops during such exposure is thus termed chilling injury in order to discriminate it from injury caused by freezing. Survival was assessed by monitoring the males' status 2 h after the end of exposure and then after 1, 2, 3 and 7 days. Three categories were distinguished: (a) *fit*, rapid and coordinated crawling; (b) *injured*, slow uncoordinated crawling or movement; (c) *dead*, no response to stimulation with a fine paintbrush. The injections of either blank solution (2 µL of injection buffer) or RNAi solution (2 µL of *Pahsp70* dsRNA) were performed two days prior to survival test. There was no difference in heat- and cold-survival between blank-injected individuals and untreated controls (data not shown).

Cross-tolerance is a process when acclimatory response to one stressful event (for instance, the synthesis of Hsps in response to heat shock) affords individuals resistance to some other stressor applied subsequently (for instance, the exposure to cold). We assessed the capacity for cross-tolerance in males of *P. apterus* by exposing them to a mild heat shock (+41°C for 1 h), then allowing them to recover at 25°C for 30 min and immediately exposing them to −5°C for 5 days. Survival was checked as described above.

## Results

### Identification of Hsp70 and Hsc70 homologues in *P. apterus*

Two cDNA fragments were sequenced. The first one was 1460 bp-long, encoded for 409 amino acids and contained 230 bp-long 3′-UTR. The amino acid translation of this fragment exhibited the highest identity and similarity (78% and 90%, respectively) to 70 kDa inducible heat shock protein from the moth, *Sesamia nonagrioides*, as well as substantial homology with 70 kDa heat shock proteins from many other insect species, including *D. melanogaster* (Hsp70B, 75% identity, 87% similarity). The second fragment was 1581 bp-long, encoded for 347 amino acids and contained 547 bp-long 3′-UTR. The amino acid translation showed substantial identity and similarity to 70 kDa heat shock cognates from many different organisms, *e.g.* the moth *Manduca sexta* (91% and 97%), the bee *Megachile rotundata* (90% and 97%), and the mosquito *Aedes aegypti* (88% and 97%). Based on the structural homology with known proteins from other insects and also based on the expression responses to the heat shock stimulus, the fragments were named *Pahsp70* (inducible form) and *Pahsc70* (cognate form) and the sequences were deposited in NCBI GenBank under accession numbers FJ386397 and FJ386398, respectively.

### Heat shock response and its RNAi suppression

**Figure 1:**
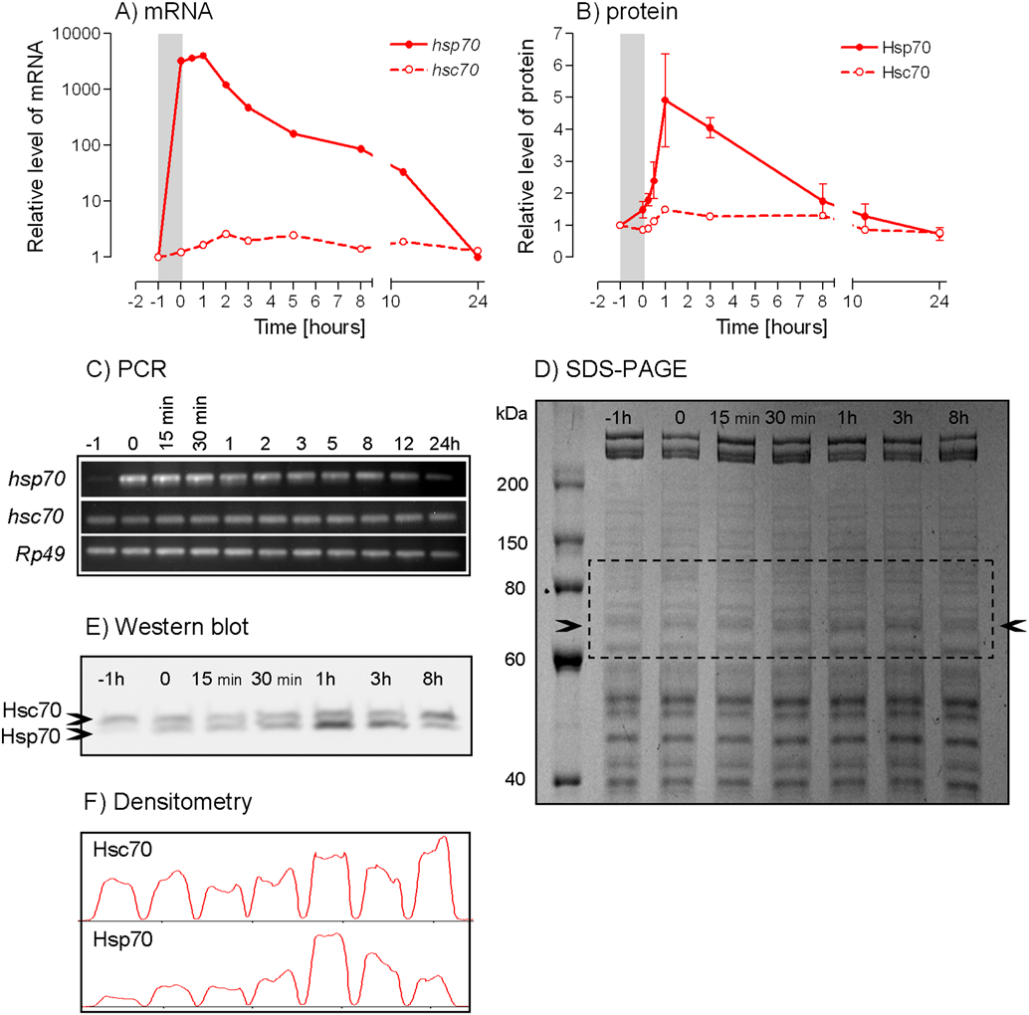
Heat shock-induced up-regulation of PaHsp70 and PaHsc70 expressions. Males of *Pyrrhocoris apterus* were exposed to +41°C for 1 h (shaded column) and then allowed to recover at 25°C. (A) Relative levels of *Pahsp70* and *Pahsc70* mRNAs in the fat body were measured by qPCR (quantitative real–time PCR with *Rp49* serving as a reference gene). The fat bodies (5 per each sample) were dissected prior to heat shock (time −1 h), at the end of it (time 0 h) and during recovery (up to time 24 h). (B) Protein levels were based on the results of Western blot hybridization. Whole procedure of electrophoresing, blotting, hybridization and densitometry was replicated three times for each sample. Data points are means±S.E.M. (C) An example of the results of standard PCR amplification with 25 cycles (note that a different method, *i.e.* q PCR was used to quantify the abundance of mRNA transcripts). The products were separated on 2% agarose and stained by ethidium bromide. It documents the temporal pattern of up-regulation of *Pahsp70* mRNA in contrast to relatively stable levels of *Pahsc70* an *Rp49* mRNAs. (D) An example of SDS-PAGE shows equal loading of proteins (20 µg). The up-regulation of PaHsp70 was only weakly detectable (arrowheads). (E) The dashed-line rectangle area of SDS-PAGE is shown after Western blotting. The mouse monoclonal anti-Hsp70 primary antibody (clone BRM-22, Sigma) recognized both PaHsp70 and PaHsc70 proteins. Note clear up-regulation of PaHsp70 at times 1 h and 3 h in contrast to relatively stable signal of PaHsc70. (F) An example of Western blot signal quantification using Quantiscan (Biosoft) densitometry.

After a mild heat shock (+41°C/1 h), the males of *P. apterus* did not fall into heat coma and no visible signs of heat-induced injury were seen. Nevertheless, this heat shock caused considerable up-regulation of *Pahsp70* mRNA levels in the fat body tissue (Fig. 1A, C), and a similar response was observed in whole body preparations (data not shown). Massive transcription of *Pahsp70* gene started already during the heat stimulus. According to qPCR quantification, the mRNA levels increased 3243-fold during the heat stimulus (a difference between the times −1 h and 0 h). The maximum level was 4038-fold higher than the level prior to heat shock and it was reached after a 1 h recovery at 25°C. The mRNA abundance returned close to the initial level within 1 day of recovery at 25°C. The up-regulation of PaHsp70 protein displayed a slight delay after the increase of mRNA levels. It started only after the end of the heat stimulus and the maximum (4.9-fold up-regulation) was reached within 1 h after the end of the heat stimulus. Protein levels dropped back to the initial values within 12 h (Fig. 1B, D–F). Heat shock also caused a slight increase of *Pahsc70* mRNA and PaHsc70 protein levels (Fig. 1A, B, D–F). The magnitude of the cognate form up-regulation, however, was much lower than that of the inducible form. The cognate mRNA levels increased 2.6-fold and the up-regulation of protein levels (1.5-fold) was hardly detectable using our Western blot technique.

**Figure 2:**
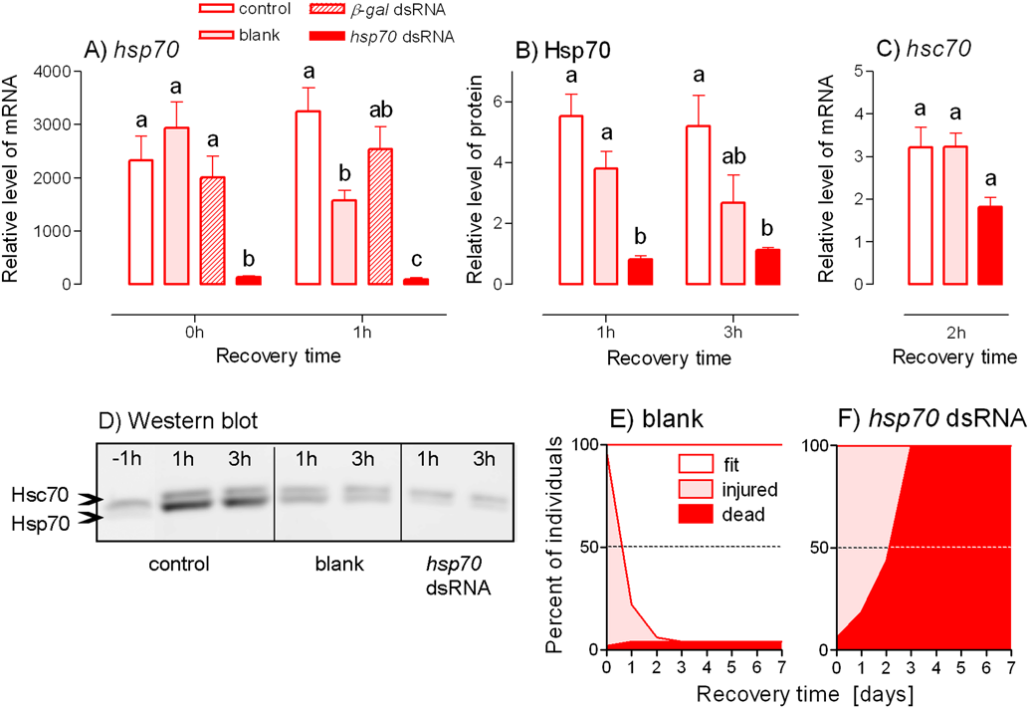
RNAi suppression of heat shock-induced up-regulation of PaHsp70 expression and its effect on survival. Relative levels of *Pahsp70* mRNA (A), PaHsp70 protein (B) and *Pahsc70* mRNA (C) were measured in the fat bodies of male *Pyrrhocoris apterus* at different times of recovery after the heat shock (+41°C for 1 h). The insects were either untreated (control) or injected two days prior to heat shock with: 2 µL of the injection buffer alone (blank); 2 µL (10 µg) of *ß-galactosidase* (*ß-gal*) dsRNA; or 2 µL (2 µg) of *Pahsp70* dsRNA. Each column is a mean±S.E.M. of 3–4 independent samples (5 fat bodies per sample). The differences in mRNA levels were assessed by ANOVA followed by Tukey's multiple comparison test at *p* = 0.05 (columns flanked by different letters differ significantly). (D) An example of Western blotting. (E, F) Survival in blank-injected (E, *n* = 39) and *Pahsp70* dsRNA-injected (F, *n* = 40) insects after a severe heat shock (+45°C for 3 h) were assessed during recovery at 25°C for 7 days. The fit insects were those showing normal, rapid and coordinated crawling; the injured insects displayed signs of heat injury, *i.e.* slow, uncoordinated crawling or movements of body appendages only; and the dead insect did not respond to stimulation with a fine paintbrush. See Fig. 1 for more information.

Injection of *ß-gal* dsRNA into the adult males had no effect on heat shock-induced up-regulation of the *Pahsp70* mRNA levels. Injecting the buffer alone (blank), although showing no effect at the end of heat shock (0 h), resulted in a slight suppression of *Pahsp70* mRNA levels measured after 1 h of recovery. In contrast, the injection of *Pahsp70* dsRNA resulted in a drastic suppression of heat-induced expression of the *Pahsp70* gene (Fig. 2A). Similarly, the up-regulation of protein levels of PaHsp70 was affected a little by the blank treatment but was practically eliminated by *Pahsp70* dsRNA injection (Fig. 2 B, D). The injection of *Pahsp70* dsRNA exhibited a non-specific cross-effect on the levels of cognate *Pahsc70* mRNA. A slight reduction of *Pahsc70* mRNA levels was detected (Fig. 2C), but this was not reflected in PaHsc70 protein levels (Fig. 2D).

Males that were exposed to a severe heat shock of +45°C/3 h were all in a heat coma (no movements) at the end of exposure and they still displayed slow and uncoordinated movements 2 h after the end of exposure. Thus, they were all classified as injured. Blank-injected individuals were able to rapidly repair heat injury and most of them (95.9%) were classified as fit by the end of a 7-day-long recovery period (Fig. 2E). In contrast, the individuals that were injected with *Pahsp70* dsRNA were not able to repair heat injury and all died within 3 days (Fig. 2F).

### Response to cold exposure and its RNAi suppression

**Figure 3:**
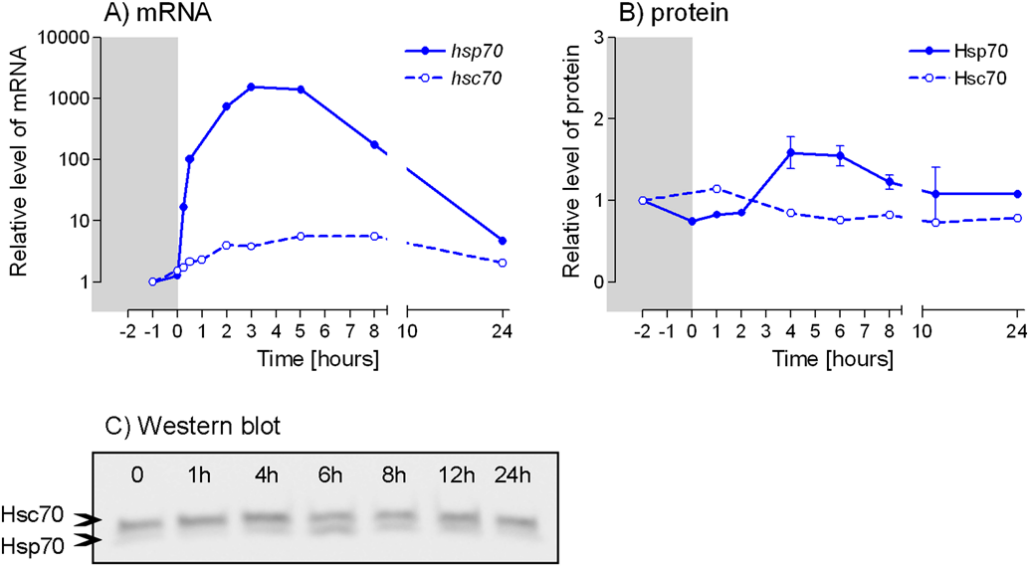
Cold exposure-induced up-regulation of PaHsp70 and PaHsc70 expressions. Males of *Pyrrhocoris apterus* were exposed to −5°C for 5 d (shaded area) and then allowed to recover at 25°C. (A) Relative levels of *Pahsp70* and *Pahsc70* mRNAs in the fat body were measured by qPCR (with *Rp49* serving as a reference gene). The fat bodies (5 per each sample) were dissected prior to cold exposure (not shown), during it (time −1 h), at the end of it (time 0 h) and during recovery (up to time 24 h). (B) Protein levels were based on the results of (C) Western blot hybridization. Data points are means±S.E.M. of three Western blot replications. See Fig. 1 for more information.

A massive transcription of *Pahsp70* gene was induced by exposing the males to a low temperature of −5°C for 5 days (Fig. 3A). The mRNA levels started to rise only after the end of cold exposure and the maximum level (1532-fold up-regulation) was reached at 3 h of recovery at 25°C. The increase in PaHsp70 protein levels was not apparent earlier than at 2 h of recovery and the maximum levels (1.6-fold up-regulation) were reached between 4–6 h of recovery (Fig. 3B, C). Although a clear up-regulation of *Pahsc70* mRNA was observed (5.6-fold increase between 6–8 h of recovery), no significant change of PaHsc70 protein levels was detected (Fig. 3A, B, C).

**Figure 4:**
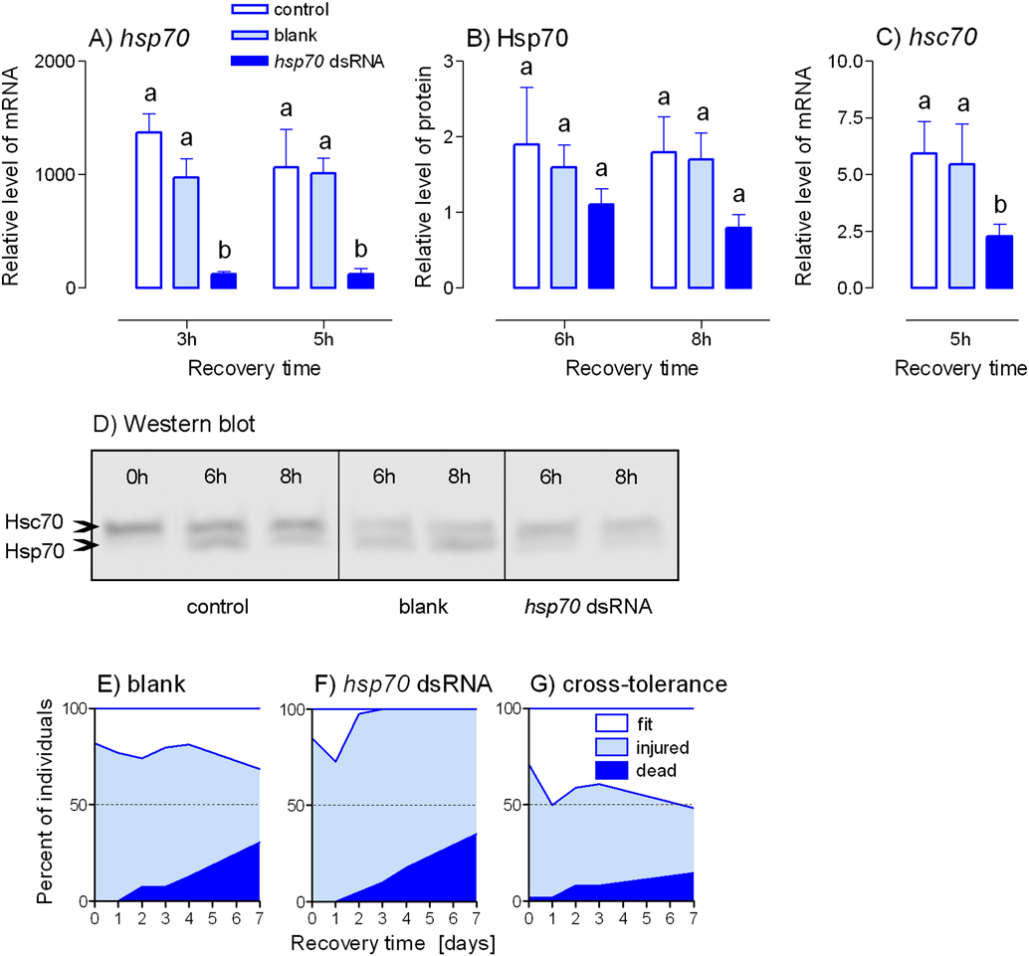
RNAi suppression of cold exposure-induced up-regulation of PaHsp70 expression and its effect on survival. Relative levels of *Pahsp70* mRNA (A), PaHsp70 protein (B) and *Pahsc70* mRNA (C) were measured in the fat bodies of male *Pyrrhocoris apterus* at different times of recovery after the cold exposure to −5°C for 5 d. The insects were either untreated (control) or injected two days prior to heat shock with: 2 µL of the injection buffer alone (blank); or 2 µL (2 µg) of *Pahsp70* dsRNA. (D) An example of Western blotting. (E, F) Survival in blank-injected (E, *n* = 49) and *Pahsp70* dsRNA-injected (F, *n* = 48) insects after the cold exposure. (G) Cross-tolerance was assesed by observing the survival in insects (*n* = 48) that were pretreated with a mild heat shock (+41°C for 1 h) prior to the cold exposure to −5°C for 5 d. See Figs. 1 and 2 for more information.

While the injection of *Pahsp70* dsRNA suppressed the cold-induced up-regulation of *Pahsp70* mRNA and PaHsp70 protein, no effect of blank injection was observed (Fig. 4A, B, D). A non-specific cross-effect of our RNAi onto the levels of cognate *Pahsc70* mRNA was confirmed (Fig. 4C).

At the end of cold exposure, all males were in a deep chill coma. They started to move within 30 min and some of them apparently recovered within 2 hours (those were classified as fit). Mortality increased gradually during the 7-day recovery period. No significant difference in mortality was found between blank- and *Pahsp70* dsRNA-injected groups (30.8% vs. 35.0% of dead individuals on day 7 of recovery period, respectively), but relatively low mortality occurred in the group tested for cross-tolerance (14.9%). The most conspicuous difference between three groups, however, appeared in the number of individuals that were able to repair chilling injury. While 37.7% of blank-injected males fully recovered after the cold exposure and were fit, none of the *Pahsp70* dsRNA-injected males was fit on day 7 (Fig. 4E, F). In the group of males tested for cross-tolerance, 51.6% of males were fit by the end of the 7-day recovery period (Fig. 4G).

## Discussion

### Up-regulation responses to heat shock and cold exposure

We cloned and sequenced two partial cDNAs encoding structural homologues of inducible and cognate forms of the proteins belonging to the Hsp70 family in *P. apterus*. Functional homology was verified by assessing the transcriptional response of the genes and up-regulation of corresponding proteins after heat shock stimulus. The temporal pattern and magnitude of mRNA and protein responses were characteristic for inducible and cognate forms of Hsp70 [41]. While very fast and massive increase of mRNA levels, followed by 1 h-delayed increase of protein levels, were observed for the inducible form (PaHsp70), relatively small increases of mRNA and protein were detected for the cognate form (PaHsc70).

We observed a significant up-regulation of PaHsp70 in response to cold exposure to −5°C for 5 days. It is not surprising to see the Hsp70 up-regulation in response to cold as the cold denaturation of proteins is a general phenomenon [42], [43] and, in turn, partially denatured or missfolded proteins are potent triggers of rapid Hsp70 accumulation [15], [44]. The response to cold differed from the response to heat stimulus in that the accumulation of mRNA transcripts of the *Pahsp70* gene started only after the end of cold exposure, *i.e.* during the recovery at 25°C, and the expression of PaHsp70 protein was shifted accordingly. Similar temporal pattern of Hsp70 cold-induced response was observed in fruit flies [17], [28].

There was a discrepancy between a large effect of heat/cold stimuli on the *Pahsp70* gene (up-regulation of mRNA levels by more than 3 orders) but a small effect on the PaHsp70 protein levels (up-regulation by less than 1 order). It is known that mRNA levels are not always perfectly mirrored by the protein levels [26]. In addition, we used relative rather than absolute quantification methods for both mRNA and protein levels, which does not allow drawing precise stochiometric conclusions.

### RNAi suppression of the up-regulation responses

Our RNAi (injection of *Pahsp70* dsRNA) caused a significant reduction of the up-regulation responses to both heat and cold stimuli. The suppression was not complete when assessed at the mRNA level. The levels of *Pahsp70* mRNA still increased approximately 100-fold in the *Pahsp70* dsRNA-treated individuals after heat or cold stimulus. Such increase, however, was dramatically lower than that in controls, where increases by 3 orders of magnitude were seen. The up-regulation responses were completely eliminated at the protein level. Indeed, the effectiveness of RNAi that we achieved was sufficient to clearly change the phenotype of *P. apterus* adults, which was proved by their complete loss of heat tolerance. None of the insects that were injected with *Pahsp70* dsRNA survived after the heat shock while 96% of blank-injected insects fully recovered and were fit. A similar loss of heat tolerance after RNAi, which was directed against the expression of Hsp70, was reported in diapausing pupae of *S. crassipalpis* [8]. The effect of RNAi on cold tolerance was less pronounced than its effect on heat tolerance. Our RNAi did not change cold-induced mortality. It significantly did affect, however, the number of insects that were able to repair their chilling injury and fully recover from cold stress. While more than 1/3 of the blank-injected individuals could be classified as fit (capable of rapid, coordinated movement, capable of successful mating), none of the *Pahsp70* dsRNA-treated individuals fully recovered after the cold stress. Interestingly, no visible signs of chilling injury were detected in approximately ¼ of the *Pahsp70* dsRNA-treated individuals at day 1 of recovery from cold stress (see Fig. 4B). Later, however, their chilling injuries became apparent, probably as a result of insufficient repair. Because the injured individuals should be considered as dead in ecological terms, we argue that our RNAi clearly demonstrated that suppression of PaHsp70 results in a loss of cold tolerance.

The injection of *Pahsp70* dsRNA resulted in relatively small non-specific suppression of the *Pahsc70* mRNA levels, which may be explained by high homology of *Pahsp70* and *Pahsc70* sequences. The region of *Pahsp70* sequence located near the 3′-end was selected as a template for dsRNA synthesis for its relatively low homology. But still, 76.8% amino acids of this region were identical in the inducible and cognate forms of PaHsp70. Since the non-specific suppression of the *Pahsc70* mRNA levels was relatively low (ca. 2-fold), and that of the protein levels was below limits of detection, we suppose that the observed phenotypic changes were related mainly to strong specific suppression of PaHsp70 mRNA and protein levels.

### Cross-tolerance

In our cross-tolerance experiment, males of *P. apterus* were first exposed to a mild heat shock in order to trigger the up-regulation of Hsp70 which, in theory, positively affects survival of subsequent cold stress. Indeed, the heat shock-pretreated males showed higher cold tolerance than blank-injected males. Cross-tolerance experiments were earlier conducted with fruit flies but ended with variable results. It was observed that survival of *D. melanogaster* larvae after a cold treatment was dramatically improved by a mild heat shock just before the cold shock [27], but other studies failed to find positive effect of heat pretreatment in larvae [45], and in adults of *D. melanogaster* [28]. Selection of adult flies for increased survival after heat shock for 21 generations resulted in improved survival after cold shock in selected flies [46]. Such diversity of results can be explained by high sensitivity of cross-tolerance assays to details of experimental design. Sub- or supra-optimal dose of heat pretreatment could be insufficient to elicit the cross-tolerance effect or damaging itself, respectively. The rapid dynamism of mRNA and protein levels during and after the heat shock suggests that the duration of the recovery period after the heat pretreatment may be of crucial significance. Although accumulation of Hsp70 was measured in some of the above mentioned studies [27], [28], it is not possible to conclude from these data (or from our data presented in Fig. 4G) whether it is the synthesis of Hsps or some other physiological change occurring during heat pretreatment which confers higher cold tolerance on the pretreated individuals.

### Up-regulation of PaHsp70 as a component of a complex cold tolerance adaptation

Low temperature affects many different structures and processes simultaneously and, therefore, insect cold tolerance is a highly complex adaptation and must be explained as a combination of different mechanisms [1], [47], [48]. This paradigm was fully supported by our previous investigation into the mechanisms of cold tolerance in *P. apterus* which increases seasonally during autumn. Upon entrance into diapause during peak of summer [37], [49], [50], reproduction ceases and metabolic pathways are redirected toward building up of reserves [51] and enhanced stress tolerance [34], [35]. During the autumn cold acclimation, the temperature of crystallization of body fluids drops to approximately −17°C [30], [33], biological membranes undergo restructuring [7], [31], cryoprotectants sorbitol and ribitol are accumulated [33], [52], and ion homeostasis and electrochemical gradients across membranes are protected [36], [53]. We suggest that the accumulation of PaHsp70 also contributes to this complex cold tolerance syndrome in *P. apterus*. Its significance was attested in non-diapause males. Non-diapause insects, however, do not express the whole battery of cold tolerance mechanisms (as described above) and, consequently, posses much lower capacity for cold acclimation and much lower level of cold tolerance than diapause insects [32]. It remains to be tested what the relative importance of Hsp70 up-regulation is on the backround of other potent cold tolerance adaptations in cold-hardy diapause insects. Higher rates of constitutive expression of Hsps were often observed in diapause insects [8], dormant invertebrates [54]–[56]; and also in dormant embryos of fish [57]. High levels of Hsps during dormancy may represent an anticipatory protection against a variety of environmental insults and/or may participate in the regulation of dormancy state.
